# Soluble Expression of Human Leukemia Inhibitory Factor with Protein Disulfide Isomerase in *Escherichia coli* and Its Simple Purification

**DOI:** 10.1371/journal.pone.0083781

**Published:** 2013-12-16

**Authors:** A. Song Jung, Bon-Kyung Koo, Seon-Ha Chong, Kyunhoo Kim, Dong Kyu Choi, Thu Trang Thi Vu, Minh Tan Nguyen, Boram Jeong, Han-Bong Ryu, Injune Kim, Yeon Jin Jang, Robert Charles Robinson, Han Choe

**Affiliations:** 1 Department of Physiology and Bio-Medical Institute of Technology, University of Ulsan College of Medicine, Seoul, South Korea; 2 Graduate School of Medical Science and Engineering Korea Advanced Institute of Science and Technology, Daejeon, South Korea; 3 Institute of Molecular and Cell Biology, A*STAR (Agency for Science, Technology and Research), Biopolis, Singapore, Singapore; 4 Department of Biochemistry, National University of Singapore, Singapore, Singapore; Centro Nacional de Biotecnologia - CSIC, Spain

## Abstract

Human leukemia inhibitory factor (hLIF) is a multifunctional cytokine that is essential for maintaining the pluripotency of embryonic stem cells. hLIF may be also be useful in aiding fertility through its effects on increasing the implantation rate of fertilized eggs. Thus these applications in biomedical research and clinical medicine create a high demand for bioactive hLIF. However, production of active hLIF is problematic since eukaryotic cells demonstrate limited expression and prokaryotic cells produce insoluble protein. Here, we have adopted a hybrid protein disulfide isomerase design to increase the solubility of hLIF in *Escherichia coli*. Low temperature expression of hLIF fused to the b'a' domain of protein disulfide isomerase (PDIb'a') increased the soluble expression in comparison to controls. A simple purification protocol for bioactive hLIF was established that includes removal of the PDIb'a' domain by cleavage by TEV protease. The resulting hLIF, which contains one extra glycine residue at the N-terminus, was highly pure and demonstrated endotoxin levels below 0.05 EU/μg. The presence of an intramolecular disulfide bond was identified using mass spectroscopy. This purified hLIF effectively maintained the pluripotency of a murine embryonic stem cell line. Thus we have developed an effective method to produce a pure bioactive version of hLIF in *E. coli* for use in biomedical research.

## Introduction

Human leukemia inhibitory factor (hLIF), also known as differentiation-stimulating factor (D factor) or melanoma-derived LPL inhibitor (MLPLI), is a cytokine that demonstrates multiple effects on cells, human physiology, and disease [1,2]. hLIF is absolutely required for maintaining the stemness of embryonic stem cell (ESC) lines [3-6]. LIF-deficient mice demonstrate difficulties in blastocyst implantation [7], which suggests that administration of hLIF may aid the implantation rate of women displaying some forms of infertility [8]. LIF also inhibits growth and promotes differentiation in murine myeloid leukemia M1 cells, the property from which its name is derived [9]. Furthermore, LIF regulates hematopoiesis, bone metabolism, neural development, embryogenesis, and inflammation [10-12].

The efficient production of hLIF has been continuously pursued because of its uses in biomedical research and clinical medicine. The expression of LIF in eukaryotic cells has been reported [13,14], although the yields are low. *Escherichia coli* (*E. coli*) produces better expression, however the protein misfolds and aggregates to form inclusion bodies (IBs) in this expression host [15]. In general, the solubilization of IBs requires high concentrations of denaturants, such as urea or guanidine hydrochloride, and subsequent refolding via the removal of the denaturants. In many cases the overall yield of biologically active protein from IBs is low [16]. Therefore, other methods to increase the soluble expression of hLIF in *E. coli* have been explored. Glutathione S-transferase (GST) and thioredoxin (Trx) have been reported either as tagged or independently expressed proteins to aid solubility and improve yields. However these methods suffer from other issues, such as the tag was not removed or the levels of endotoxin were high or not tested, which are important considerations for maintaining biological activity *in vivo* [17-21].

In this study, we observed that protein disulfide isomerase (PDI) significantly improves the solubility of hLIF as a fusion protein in *E. coli*. Low temperature expression dramatically increases the solubility of hLIF as a hybrid protein with b'a' domain of the PDI (PDIb'a'), which is a small functional fragment of PDI. We also established a simple protocol for purifying bioactive hLIF. The disulfide bond and biological activity of hLIF were confirmed using mass spectroscopy and ESC culture assays, respectively. 

## Materials and Methods

### Construction and expression of His8-hLIF, PDIb'a'-hLIF, and PDI-hLIF

 The DNA sequence encoding the 180 amino acids of mature hLIF (NCBI reference sequence: NP_002300.1) was synthesized (Bioneer, Daejeon, Korea). The codons were optimized for *E. coli* in order to improve the expression of mature hLIF (Figure S1 in File SI). The DNA encoding a tobacco etch virus (TEV) protease recognition site (TEVrs; ENLYFQˇG) was inserted at the N-terminus of hLIF in order to cleave the tags from hLIF. *att*B1 (5'-ggggacaagtttgtacaaaaaagcaggcttc-3') and *att*B2 (5'-acccagctttcttgtacaaagtggtcccc-3') were also inserted at the 5'- and 3'-ends, respectively, to recombine the *att*P sites in the donor vector. The synthesized DNA was subcloned into pDONOR207 by BP recombination [22], and the resulting entry vector was named pENTR-hLIF. To obtain expression vectors, hLIF from pENTR-hLIF was transferred to pDEST-His8, pDEST-PDI, and pDEST-PDIb'a' [23] by LR recombination [22]. These BP and LR recombination procedures (Gateway system; Invitrogen, Carlsbad, CA) introduce 57 nucleic acids (ggtggtctggttccgcgtggttccatcacaagtttgtacaaaaaagcaggcttc, which encodes 19 amino acids, GGLVPRGSITSLYKKAGFM) between His8 and TEVrs. However, these extra amino acids together with the His8 tag were later removed by TEV protease treatment leaving a single extra amino acid (Gly) at the C-terminus. The gene for the PDIb'a' domain was amplified from the *PDI* gene using PCR and the Phusion flash high-fidelity PCR master mix (Finnzymes, Espoo, Finland). All constructed sequences were confirmed by DNA sequencing (Macrogen, Daejeon, Korea). 

The three expression vectors were transformed into competent *E. coli* BL21 (DE3) host cells. The transformants were cultured at 37°C in LB medium containing 25 μg/ml kanamycin or 50 μg/ml ampicillin (as appropriate), and the target protein expression was induced using 0.5 mM isopropyl-β-D-thiogalactoside (IPTG) when the optical density at 600 nm (OD_600_) reached 0.6 au. After the addition of IPTG, the culture was maintained at 37°C for 4 hours or, alternatively, maintained at 18°C for 18 hours in order to increase protein solubility. All expression levels were analyzed using sodium dodecyl sulfate-polyacrylamide gel electrophoresis (SDS-PAGE) with 10% Tris-glycine gel.

### Purification of His8-hLIF

Cells were harvested by centrifugation at 3500 x g for 30 minutes at 4°C, and the cell pellets were resuspended in lysis buffer LA (20 mM Tris-HCl, pH 8.0, 300 mM sodium chloride, 5% glycerol (v/v), 1 mM phenylmethylsulfonyl fluoride (PMSF)) at a 10 ml/g ratio. The lysate was sonicated 20 times on ice for 30 seconds with 30 second interruptions using an ultrasonic cell disruptor JY99-IIDN set at 1500 W (Ningbo Scientz Biotechnology, Guangdong, China), then the supernatant was clarified by centrifugation at 27,000 x g for 30 minutes at 4°C and filtered through a 0.45 μm syringe filter PTFE (CHEMCOKOREA, Cheongwon, Korea). The overexpressed His8-hLIF fusion protein was initially purified using immobilized metal ion affinity chromatography (IMAC). The filtered lysate was loaded onto a 5 ml HisTrap HP column (GE Healthcare, Piscataway, NJ) that had been equilibrated with buffer A (20 mM Tris-HCl, pH 8.0, 300 mM sodium chloride, 5% glycerol (v/v)). The column was extensively washed with 8–10 column volumes (CVs) of buffer B (20 mM Tris-HCl, pH 8.0, 300 mM sodium chloride, 5% glycerol (v/v), 150 mM imidazole) to remove any nonspecifically bound proteins. Protein samples were eluted with 6 CVs of buffer C (20 mM Tris-HCl, pH 8.0, 300 mM sodium chloride, 5% glycerol (v/v), 1 M imidazole). The eluted His8-hLIF fusion protein was proteolytically digested using a homemade TEV protease 1:1 (w/w) supplemented by 1 mM dithiothreitol (DTT; reaction performed at 30°C for 3 hours).

Following proteolytic cleavage, hLIF was subjected to an additional IMAC step to remove the tag and any uncleaved protein. Here, the cleaved protein solution was placed in a dialysis membrane (3500 Da molecular weight cutoff; Viskase, Darien, IL) and dialyzed twice against buffer A at a 1:100 (v/v) ratio for 12 hours at 4°C to remove DTT and imidazole; then, the cleaved fusion protein was loaded onto a 5 ml HisTrap HP column that had been equilibrated with buffer A. The column was washed with 2 CVs of buffer A. Cleaved hLIF was eluted with 2 CVs of buffer D (20 mM Tris-HCl, pH 8.0, 300 mM sodium chloride, 5% glycerol (v/v), 100 mM imidazole), and then the cleaved His8 tag and TEV protease were eluted with elution buffer C. Fractions containing the purified hLIF protein were dialyzed against buffer E (20 mM Tris-HCl, pH 8.0, 200 mM sodium chloride, 5% glycerol (v/v)) to decrease the concentration of sodium chloride and remove imidazole. All protein concentrations were determined using the Bradford method [24] with bovine serum albumin (BSA) employed as the standard.

### Purification of PDIb'a'-hLIF

Cells were harvested as described above, and then the pellets were resuspended in lysis buffer LB (20 mM Tris-HCl, pH 7.0, 5% glycerol (v/v), 0.5 mM ethylenediaminetetraacetic acid (EDTA), 1 mM PMSF). The PDIb'a'-LIF lysate was sonicated as for His8-LIF. The supernatant was filtered through a 0.45 μm syringe filter PTFE (CHEMCOKOREA) and loaded into a 5 ml HiTrap SP column (GE Healthcare) that had been pre-equilibrated with buffer F (20 mM Tris-HCl, pH 7.0, 5% glycerol (v/v), 0.5 mM EDTA). The supernatant was then washed with 2 CVs of buffer F to remove any nonspecifically bound proteins, and then a linear gradient of sodium chloride was applied to a maximum of 1 M sodium chloride in buffer F. The protein eluted at between 25–45% of the sodium chloride gradient. The pool of eluted fractions was dialyzed in buffer G (20 mM Tris-HCl, pH 8.0, 5% glycerol (v/v), 0.5 mM EDTA), and then loaded into a 5 ml HiTrap Q HP column (GE Healthcare) that had been equilibrated with buffer G. The column was washed with 2 CVs of buffer G, and then a linear gradient of sodium chloride was applied to a maximum of 1 M sodium chloride in buffer G. The protein eluted at between 45–60% of the sodium chloride gradient. The pool of eluted fractions was treated overnight at room temperature using 1 μg TEV protease [25] in order to cleave 10 μg of fusion protein 10:1 (w/w) in the presence of 1 mM DTT.

Following proteolytic cleavage, the reaction mixture was concentrated to 1 ml using an Amicon Ultra-15 centrifugal filter unit (Millipore, Billerica, MA) and was loaded onto a HiLoad 16/60 Superdex-75 HR column (GE Healthcare) that had been equilibrated with buffer A. The hLIF clearly separated from the PDIb'a' tag eluted in approximately 75 ml of the retention volume. To remove the residual TEV protease, the eluent from Superdex 75 HR was loaded into a 5 ml HisTrap HP column (GE Healthcare) that had been equilibrated with buffer A and washed with 2 CVs of binding buffer. Cleaved hLIF was eluted with 2 CVs of buffer D. Fractions containing the purified hLIF protein were dialyzed in the buffer E to decrease the concentration of sodium chloride and remove imidazole. 

### Purification of TEV protease

The plasmid containing the mutated TEV protease gene, *pRK793* [25], was transformed into BL21(DE3) cells. One colony was selected from LB agar plates containing ampicillin (50 μg/ml) and was subsequently grown in LB broth with antibiotic at 37°C overnight. Then, 0.5% of the cells were inoculated in 1 L LB broth containing ampicillin (50 μg/ml) and incubated at 37°C with shaking at 180 rpm until the OD_600_ reached 0.4–0.6 au. The TEV protease was induced by the addition of 0.5 mM IPTG. During TEV protease expression, the temperature was decreased to 30°C and allowed to incubate for 6 hours or overnight. The cells were harvested by centrifugation at 3500 x g for 20 minutes at 4°C.

The harvested cells were resuspended in lysis buffer LC (20 mM Tris-HCl, 500 mM sodium chloride, 1% Triton X-100 (v/v), 5% glycerol (v/v), 1 mM PMSF) (10 ml per 1 g cells) and sonicated 20–30 times on ice using 30-second pulses and 30-second interruptions using an ultrasonic cell disruptor JY99-IIDN (Ningbo Scientz) at 1500 W. The lysate was centrifuged at 27,000 x g for 20 minutes at 4°C, and then the supernatant was filtered through a 0.45 μm syringe filter PTFE (CHEMCOKOREA). The supernatant was loaded into a HisPrep FF 16/10 column (GE Healthcare) that had been equilibrated with the buffer H (20 mM Tris-HCl, pH 8.0, 500 mM sodium chloride, 5% glycerol (v/v)). The column was washed with 5 CVs of the buffer I (20 mM Tris-HCl, pH 8.0, 500 mM sodium chloride, 5% glycerol (v/v), 60 mM imidazole) to remove any nonspecifically bound proteins except TEV protease. TEV protease was eluted using 2 CVs of the buffer J (20 mM Tris-HCl, pH 8.0, 500 mM sodium chloride, 5% glycerol (v/v), 1 M imidazole), and 4 mM DTT was immediately added to prevent precipitation. The protein was concentrated to 10–15 ml using an Amicon Ultra-15 centrifugal filter unit (Millipore) and loaded into a HiPrep 16/10 Desalting column (GE Healthcare) to change to buffer K (50 mM Tris-HCl, pH 8.0, 1 mM EDTA, 0.1% Triton X-100 (v/v), 10% glycerol (v/v)). The TEV protease-containing fractions were collected and an additional 5 mM DTT and 50% glycerol (v/v) were added. The final product was stored in aliquots at -80°C (1 unit of the purified TEV protease cleaves ≥ 85% of 2 μg tag-fused protein when incubated overnight at 18°C; the activity of the purified TEV protease was 7 units/μl). 

### Electrophoresis and silver staining

All purified proteins were analyzed using SDS-PAGE [26]. Protein samples were denatured in 5 X sample buffer (312.5 mM Tris-HCl, pH 6.8, 50% glycerol (v/v), 5% SDS (w/v), 0.05% bromophenol blue (w/v)) containing 5% β-mercaptoethanol (v/v) for 10 minutes at 100°C. Samples were loaded onto 10% polyacrylamide gels. Protein bands were developed using Coomassie brilliant blue R-250 stain (AMRESCO, Solon, OH) and analyzed using ImageJ software (NIH, Bethesda, MD). The expression levels were calculated as the percentage of overexpressed target protein within the total protein in *E. coli*. Solubility was calculated as the percentage of soluble protein. The final purified hLIF was stained and confirmed using the Silver Stain Plus kit (Bio-Rad Laboratories, Hercules, CA) according to the manufacturer’s instructions. The reaction was stopped using 5% acetic acid (v/v) for 15 minutes once the bands were clearly visible. Gels were washed with water, incubated overnight, and preserved in an aqueous solution containing 5% glycerol (v/v) and 0.02% sodium azide (w/v).

### Endotoxin assay

The endotoxins in the final purified hLIF protein were removed by treatment with Triton X-114 [27]. The purified hLIF was treated with 1% Triton X-114 (v/v; Sigma-Aldrich, St. Louis, MO) and gently mixed by inverting the tube. Next, the mixture was slowly shaken at 4°C for 30 minutes, kept at room temperature for 10 minutes, and then centrifuged at 15,000 x g for 10 minutes at room temperature. The supernatant was carefully transferred to clean tubes to avoid contamination with endotoxin and Triton X-114. The amount of remaining endotoxin was then determined using the quantitative Endpoint Chromogenic Limulus Amebocyte Lysate (LAL) test (Lonza, Basel, Switzerland). Fifty microliters of a 1 μg/ml sample or standard endotoxin (1.0, 0.5, 0.25, and 0.1 EU/ml) were dispensed into prewarmed (37°C) endotoxin-free microplate wells. Then, 50 μl of the LAL reagents were added to each well. The plate was incubated at 37°C for 10 minutes. After incubation, 100 μl of the prewarmed chromogenic substrate solution was added to each well and incubated for 6 minutes at 37°C. Then, 100 μl of termination reagent (25% acetic acid (v/v)) was added, and the wells were analyzed at 405 nm using a spectrophotometer. After measuring the absorbances, the endotoxin concentration was determined from the standard curve.

### Stability test

His8-hLIF was dialyzed using several buffers with different salt concentrations (Table S2 in File SI) at a 1:100 (v/v) ratio, two changes, for more than 4 hours at 4°C against a dialysis membrane (6000 Da molecular weight cut-off value; Spectrum Medical Industries, Los Angeles, CA). Each dialyzed His8-hLIF protein was centrifuged at 16,000 x g at 4°C for 5 minutes, and the supernatants were carefully transferred to clean 1.5 ml tubes and the concentrations of the supernatants were determined. The His8-hLIF, PDIb'a'-hLIF, and hLIF proteins that were purified from the cleaved fusion proteins were kept at 37°C. Protein samples were progressively collected and centrifuged at 16,000 x g for 1 minute to separate the supernatant from the precipitate and the supernatant concentrations were measured. The precipitation rate was calculated using Excel.

### Matrix-assisted laser desorption/ionization-time of flight mass spectrometry (MALDI-TOF MS) analysis

MALDI-TOF MS was used to verify the identity of the purified recombinant fusion proteins. In addition, reduced and nonreduced samples of hLIF were analyzed to confirm the disulfide bond formation. Proteins were treated with 10 mM DTT (reducing) or without DTT (nonreducing conditions) and incubated at room temperature for 10 minutes. Samples were precipitated with 10% trichloroacetic acid (v/v; Sigma-Aldrich) and incubated for 10 minutes on ice, and then the supernatants were removed after centrifugation at 16,000 x g at 4°C for 20 minutes. The samples were resuspended in the buffer (0.5 M Tris-HCl, pH 8.0, 5% glycerol (v/v), 100 mM sodium chloride, 1 mM EDTA, 2% SDS) containing 50 mM iodoacetamide (IAA), incubated at room temperature for 1 hour in the dark, and loaded onto a 14.4% Tris-tricine gel. The band corresponding to the recombinant fusion protein was placed in a new 1.5 ml tube, treated with 100 μl of solution 1 (200 mM ammonium hydrogen carbonate, 40% acetonitrile (ACN; v/v)), and incubated for 10 minutes at 37°C. The solution was discarded, and this process was repeated for 3–4 times to remove any dye from the gel. The gel was completely dried using a vacuum concentrator (Eppendorf, Hamburg, Germany), treated with 10 μl of trypsin (20 μg/ml), and dissolved overnight at 37°C in solution 2 (40 mM ammonium hydrogen carbonate, 9% ACN (v/v), 1 mM HCl). After centrifugation at 16,000 x g for 10 minutes, the supernatant was transferred to a new 1.5 ml tube and spotted with 10–20 mg/ml matrix (α-cyano-4-hydroxycinnamic acid (CHCA); Sigma-Aldrich) in solution 3 (70% ACN (v/v), 0.1% trifluoroacetic acid (v/v)) in order to form a 1:1 ratio on the MALDI-TOF MS plate. The masses of the fragments were measured using a Voyager-DE STR (Applied Biosystems, South San Francisco, CA) and a 4700 MALDI-TOF/TOF (AB Sciex, Framingham, MA). Data were interpreted using Data Explorer (Applied Biosystems). The NCBI database and the Mascot peptide mass fingerprinting search program (Matrix Science, Boston, MA) were used to interpret the obtained peptide masses.

### ESC culture and Flk1^+^ cell induction

ESC maintenance and differentiation assays were performed as described by Joo et al [28]. The E14tg2a4 murine ESC (mESC) line [29] was provided by Dr. Jun K. Yamashita (Kyoto University, Kyoto, Japan). This line was cultured on 0.1% gelatin-coated dishes at a density of 1 × 10^3^ cells/cm^2^ and maintained in Glasgow minimum essential medium (GMEM; Invitrogen) supplemented with 15% fetal bovine serum (FBS), sodium pyruvate, nonessential amino acids, penicillin, streptomycin, and β-mercaptoethanol in the presence of murine LIF (positive control; Millipore), purified hLIF from His8-hLIF, purified hLIF from PDIb'a'-hLIF, or purified noncleaved His8-hLIF. The concentrations of all LIFs were 1 nM. Cell media were changed every day. For Flk1^+^ cell induction, mESCs were plated onto 0.1% gelatin-coated dishes at a density of 2 × 10^3^ cells/cm^2^ without supplemental LIF. Cell media were changed every 2 days. On day 4.5, the generation of Flk1^+^ cells was analyzed using anti-mouse Flk1 antibodies (eBioscience, San Diego, CA).

### Flow cytometry

Cells were harvested using Dissociation Buffer (Invitrogen) and resuspended in Hank's Balanced Salt Solution (HBSS) with 2% FBS at a concentration of 2 × 10^6^ cells per 100 μl. Cells were incubated for 10 minutes with anti-mouse SSEA-1 (Abcam, Cambridge, UK) or biotin-conjugated anti-mouse Flk1 (eBioscience). After washing twice in HBSS with 2% FBS, the cells were incubated with PE-conjugated streptavidin (eBioscience) or FITC-conjugated anti-mouse IgM antibody. Analyses were performed using a FACS Aria II (Becton Dickinson, Franklin Lakes, NJ), and dead cells were excluded using 7-AAD (7-Amino-actinomycin D; Invitrogen). Unstained ESCs were used to determine the background expression levels. Data were analyzed using the Flow Jo software (Tree Star, Ashland, OR).

### Quantitative real-time PCR

Total RNA was extracted using the RNeasy Mini Kit (Qiagen, Venlo, Netherlands). Reverse transcription was performed using the GoScript cDNA synthesis system (Promega, Madison, WI). The resulting cDNA was used to perform quantitative real-time polymerase chain reaction (qRT-PCR) using Fast Start SYBR Green Master (Roche Applied Science, Bavaria, Germany) in an S1000 thermal cycler (Bio-Rad Laboratories). β-actin was used as the endogenous control to normalize the expression levels. cDNA was amplified using the following primer sets: nanog, F: 5'-AGGGTCTGCTACTGAGATGCTCTG-3', R: 5'-CAACCACTGGTTTTTCTGCCACCG-3' ; Oct3/4, F: 5'-TCTTTCCACCAGGCCCCCGGCTC-3', R: 5'-TGCGGGCGGACATGGGGAGATCC-3'; nestin, F: 5'-GTCCCTTAGTCTGGAAGTGGCTAC-3', R: 5'-GAAAGGCTGTCACAGGAGTC TCAAG-3'; brachyury, F: 5'-TACATCCACCCAGACTCGCCCAATTT-3', R: 5'-AAAGCAGTGGCTGGTGATCATGCGTT-3'; β-actin, F: 5'-GCTCTTTTCCAGCCTTCCTT-3', R: 5'-CTTCTGCATCCTGTCAGCAA-3'. The primer sets for nanog and Oct3/4 were obtained from a study on induced pluripotent stem cells [30].

### Statistics

Triplicate experiments were performed, and data are expressed as the mean ± standard deviation. The student *t* test, two-tailed paired test, and analysis of variance (ANOVA) were used to analyze the data. Statistical significance was defined as *p* < 0.05.

## Results

### Plasmid construction and solubility testing

**Figure 1:**
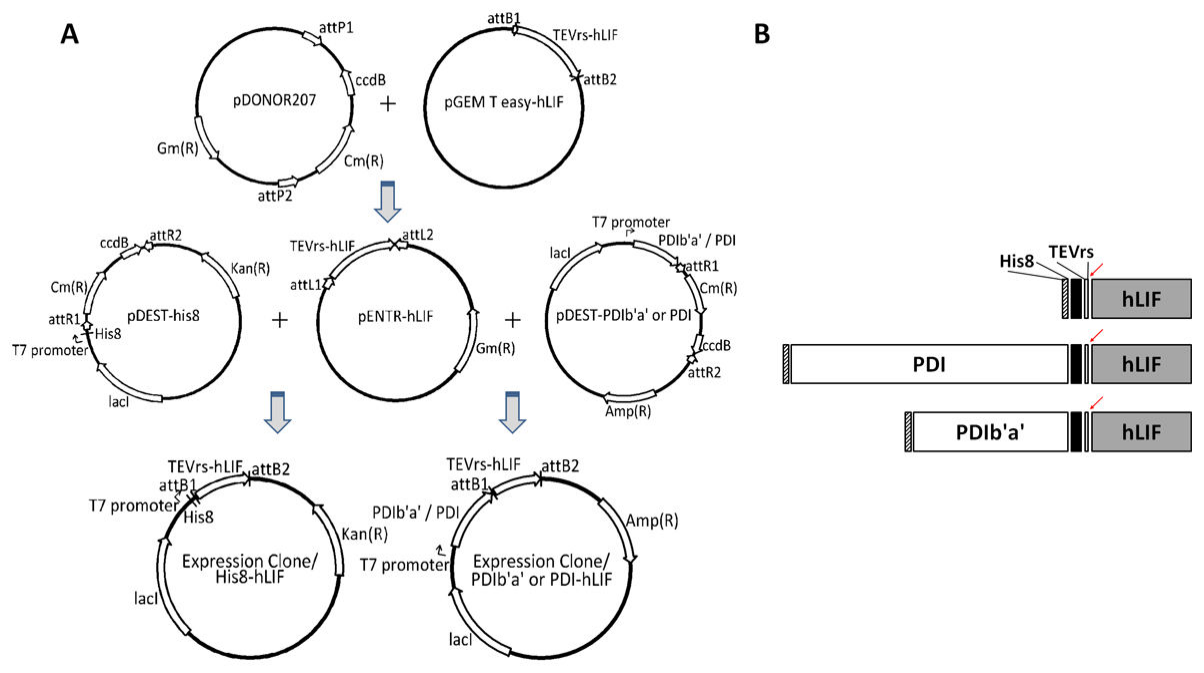
Construction and schematic representation of the His8-hLIF, PDI-hLIF, and PDIb'a'-hLIF expression vectors. (A) Vector maps of His8-hLIF, PDI-hLIF, and PDIb'a'-hLIF. Fusion protein expression in *E. coli* is controlled by the IPTG-inducible T7 promoter, and the selection markers for His8-hLIF for PDI-hLIF are kanamycin and ampicillin, respectively. (B) Schematic structure of the His8-hLIF, PDI-hLIF, and PDIb'a'-hLIF fusion proteins. The arrows indicate the recognition sites for the proteolytic cleavage of TEV protease. The black bars indicate the additional sequences that resulted from Gateway plasmid recombination. The lengths of each domain are scaled to the length ratio of each domain.

To evaluate the soluble expression of hLIF in *E. coli*, three plasmids containing hLIF with N-terminal tags were constructed: His8, PDI, and PDIb'a' (Figure 1). The genes for hLIF and PDI were codon optimized for *E. coli* expression and synthesized (Figure S1 in File SI). The encoding DNA sequence for the PDIb'a' domain was amplified from the *PDI* gene using PCR. A TEV protease recognition sequence was inserted between the N-terminal tags and hLIF to allow proteolytic cleavage of the tags during purification. The sequence-confirmed plasmids were transformed into BL21 (DE3) *E. coli* cells.

**Figure 2:**
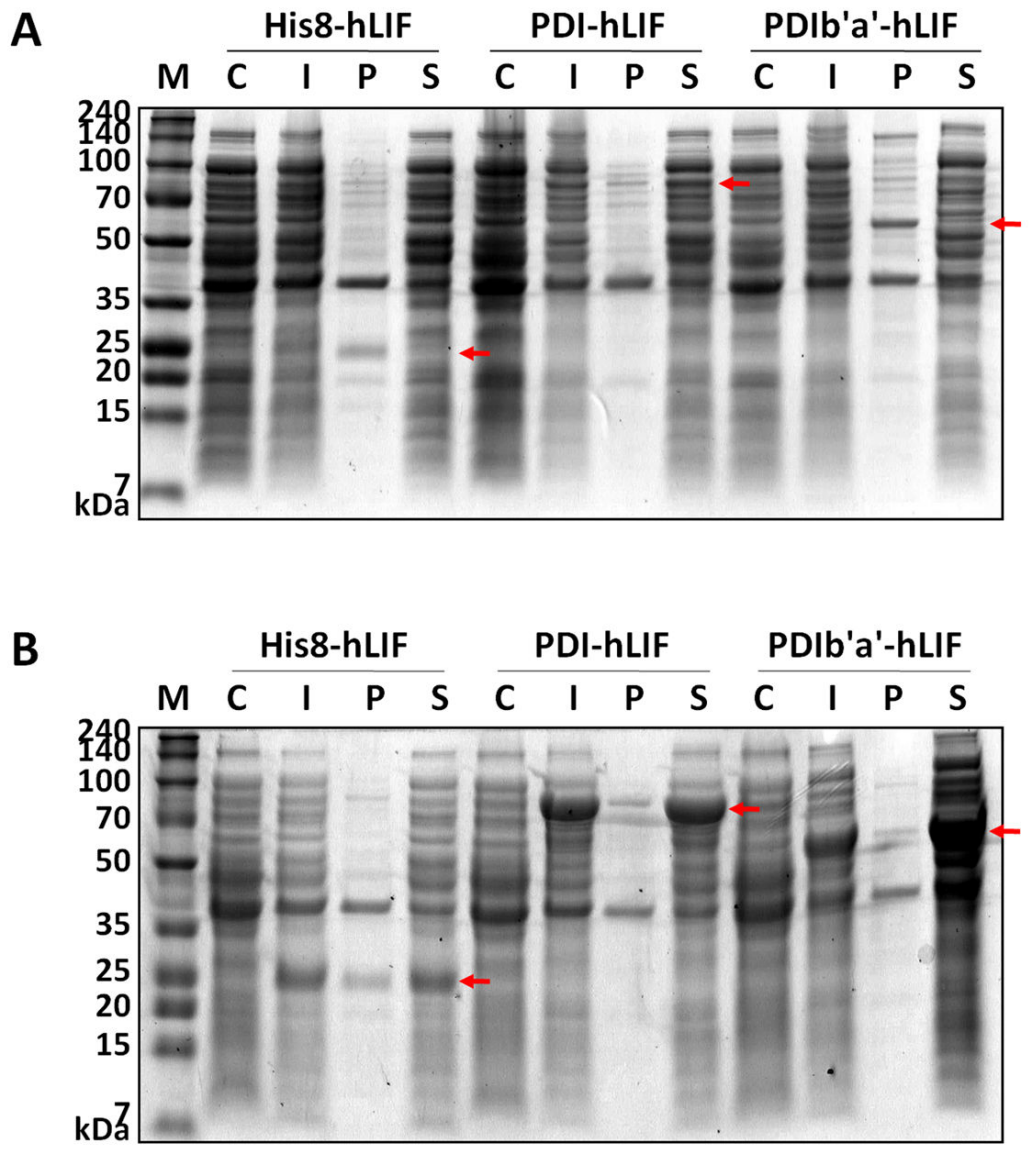
SDS-PAGE analysis of hLIF fused with three different tags in *E. coli*. Protein expression was induced by 0.5 mM IPTG at (A) 37°C and (B) 18°C. Arrows indicate the hLIF proteins fused with each tag. Equal amounts of cells were loaded into the C and I lanes. For the P and S lanes, the volume of the precipitant obtained after sonication and centrifugation was adjusted to ensure that it was obtained from the same amount of cells. 10 μl of sample was loaded. M, molecular weight size marker; C, total cellular protein before IPTG induction (control); I, total cellular protein after IPTG induction; P, insoluble cell pellet; S, soluble supernatant after sonication.

The solubility of His8-hLIF at 37°C was low (~22%), suggesting that 78% of the His8-hLIF resided in IBs (Figure 2A and Table 1). However, the solubility of PDI-hLIF was considerably higher at 97%. The PDIb'a' tag also increased the solubility of the tagged hLIF by almost 2-fold. Expression levels increased from 5.7% to 8.6% and 9.2% using PDI and PDIb'a', respectively. On lowering the expression temperature to 18°C, the solubilities of His8- and PDIb'a'-hLIF increased to 80.1% and 99.5%, respectively (Figure 2B and Table 1). Lowering the expression temperature also increased the expression levels of all three proteins by > 25%. To compare the biochemical and biological characteristics, two constructs, His8-hLIF and PDIb'a'-hLIF, were selected. PDIb'a'-hLIF was chosen over PDI-hLIF since the PDIb'a' tag is smaller, and the expression level and solubility of PDIb'a'-hLIF are also higher.

**Table 1:** Expression and solubility of hLIF determined using different tags.

	Tag	Tag size (kDa)	Fusion protein size (kDa)	Expression level (%)			Solubility (%)	
				37°C	18°C		37°C	18°C
hLIF (19.8 kDa)	His8	5.2	25.0	5.7	25.7		22.0	80.1
	PDI	59.8	79.6	8.6	29.6		97.0	95.4
	PDIb'a'	35.6	55.4	9.2	28.3		41.8	99.5

### Purification of hLIF

**Figure 3:**
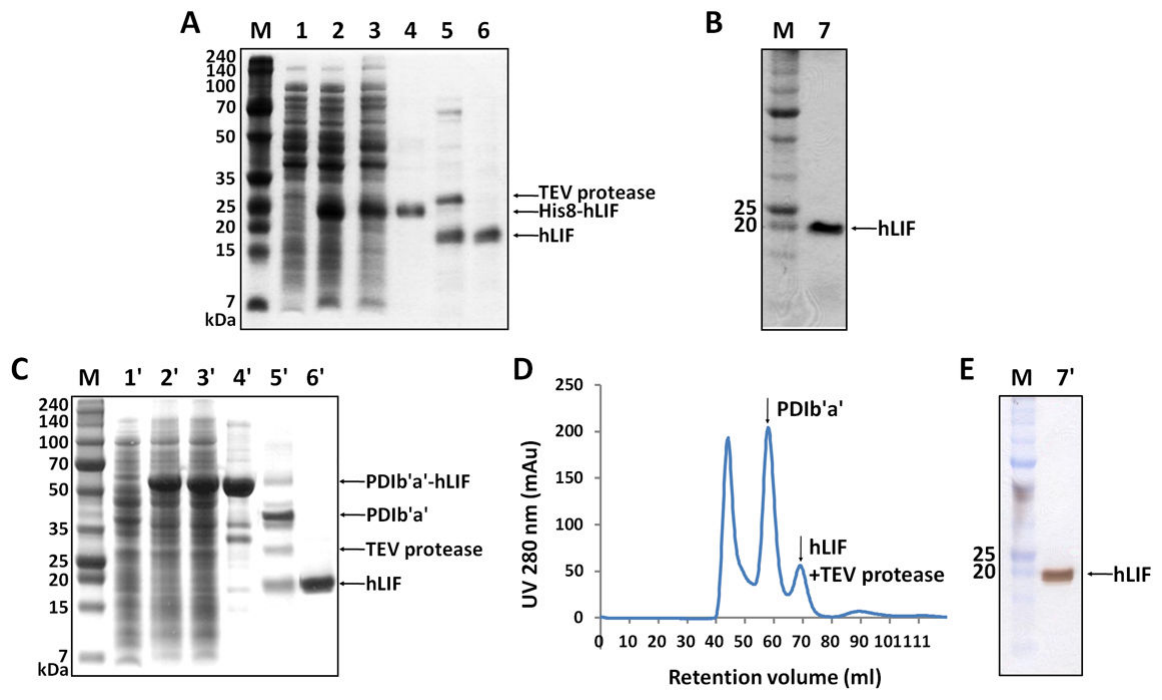
Purification of hLIF from *E. coli*. Purified (A) His8-LIF and (C) PDIb'a'-hLIF obtained from *E. coli* were analyzed using SDS-PAGE. Lanes 5 and 5' show the cleavage of His8 and PDIb'a'. M, molecular weight marker; lanes1 and 1', total cells before IPTG induction (negative control); lanes 2 and 2', total cells treated with IPTG; lanes 3 and 3', soluble fraction after sonication; lane 4, His8-hLIF fusion protein purified using HisTrap HP(25 kDa); lane 4', PDIb'a'-hLIF fusion protein purified using ion-exchange chromatography (55.4 kDa); lane 5, His8 tag cleaved using TEV protease; lane 5', PDIb'a' tag cleaved using TEV protease; lanes 6 and 6', final purified hLIF. The amount of loaded protein was individually adjusted for each lane in order to make the bands clearly visible. (B, E) Silver-stained gel from SDS-PAGE used to assess purified hLIF: 1.6 μg and 1.4 μg hLIF obtained from His8-hLIF and PDIb'a'-hLIF were loaded into lanes 7 and 7', respectively. (D) Gel filtration chromatogram of PDIb'a'-hLIF after cleavage. hLIF and PDIb'a' were separated according to size. The TEV protease eluted with hLIF.

His8-hLIF and the cleaved hLIF were purified using IMAC (Figure 3A). First, the His8-hLIF fusion protein was subjected to a Ni-Sepharose column. This step removed most of the other proteins (Figure 3A, lane 4). Purified His8-hLIF was then digested using TEV protease. To determine the optimal digestion conditions, different concentrations of sodium chloride and imidazole, several temperatures and incubation times were assessed based on the activity levels of the homemade TEV protease (Table S1 and Figure S2 in File SI). Approximately 95% of His8-hLIF was cleaved by the TEV protease 1:1 (w/w) after 3 hours at 30°C, but nonspecific cleavage was not detected. The cleavage reaction yielded 20 kDa (hLIF) and 5 kDa products (His8 tag). The subcloning method puts an additional 19 amino acids between the His8 tag and the TEV recognition site, hence the size of the His8 tag was a slightly larger than the His8 tag plus the TEV recognition site. hLIF was separated from His8 and the TEV protease using a second Ni-Sepharose column. After dialysis, ~1.8 mg hLIF was routinely obtained from 3.8 g cells (wet weight) from 1 L culture without yield optimization.

PDIb'a'-hLIF was purified from *E. coli* extracts using two ion-exchange chromatography columns (SP and Q Sepharose). The fusion protein was treated overnight at a protein:TEV ratio of 10:1 (w/w) at RT. Digestion conditions were not optimized because the fusion protein was almost completely cleaved after the first trial. Thus, PDIb'a'-hLIF digested better (protein:TEV = 10:1 (w/w)) than His8-hLIF (protein:TEV = 1:1 (w/w)) using less TEV protease. The cleaved hLIF and TEV protease were separated from the cleaved PDIb'a' tag by gel filtration (Figure 3D). To separate TEV protease from hLIF, a Ni-Sepharose column was used since the TEV protease contains an N-terminal polyhistidine tag. Approximately 1.3 mg of highly pure hLIF was obtained from 3.8 g cells (wet weight) of 1 L culture.

To assess the purity of hLIF, SDS-PAGE gels were stained using the silver staining method. Only one band was visible at 20 kDa, which is the approximate size of hLIF (Figures 3B and 3E). The endotoxin levels of the purified protein solutions were also determined using the LAL testing. The measured endotoxin concentrations of the hLIF solutions from PDIb'a'-hLIF and His8-hLIF were 0.042 and 0.046 EU/μg, respectively; these are significantly lower than 1 EU/μg, a rule-of-thumb criterion for a safe protein product.

### Low salt concentration and temporal stability

Initially, ion-exchange chromatography was used following IMAC to increase the purity of His8-hLIF. However, when the salt concentration of the sample was lowered for the ion exchange step, 94% of the His8-hLIF precipitated (Table S2 in File SI). In comparison, only 9% of PDIb'a'-hLIF precipitated using the same procedure. Several different buffer conditions were tested to determine their preventive effects on His8-hLIF precipitation, but none were successful (Table S2 in File SI). However, when the His8 tag was removed, the cleaved hLIF was soluble at low salt concentrations (data not shown). Therefore, we were able to purify hLIF from His8-hLIF.

We also measured the long-term stability of cleaved hLIF and tagged hLIF at both 37°C and room temperature. All purified proteins gradually precipitated (Figure S3 in File SI). Interestingly, His8-hLIF and hLIF cleaved from His8-hLIF at 37°C demonstrated better stability than PDIb'a'-hLIF and hLIF cleaved from PDIb'a'-hLIF at room temperature. The former hLIF demonstrated an average precipitation rate of 1.8% per day, whereas the precipitation rate of the latter was 3.2% per day.

### MS analysis

**Figure 4:**
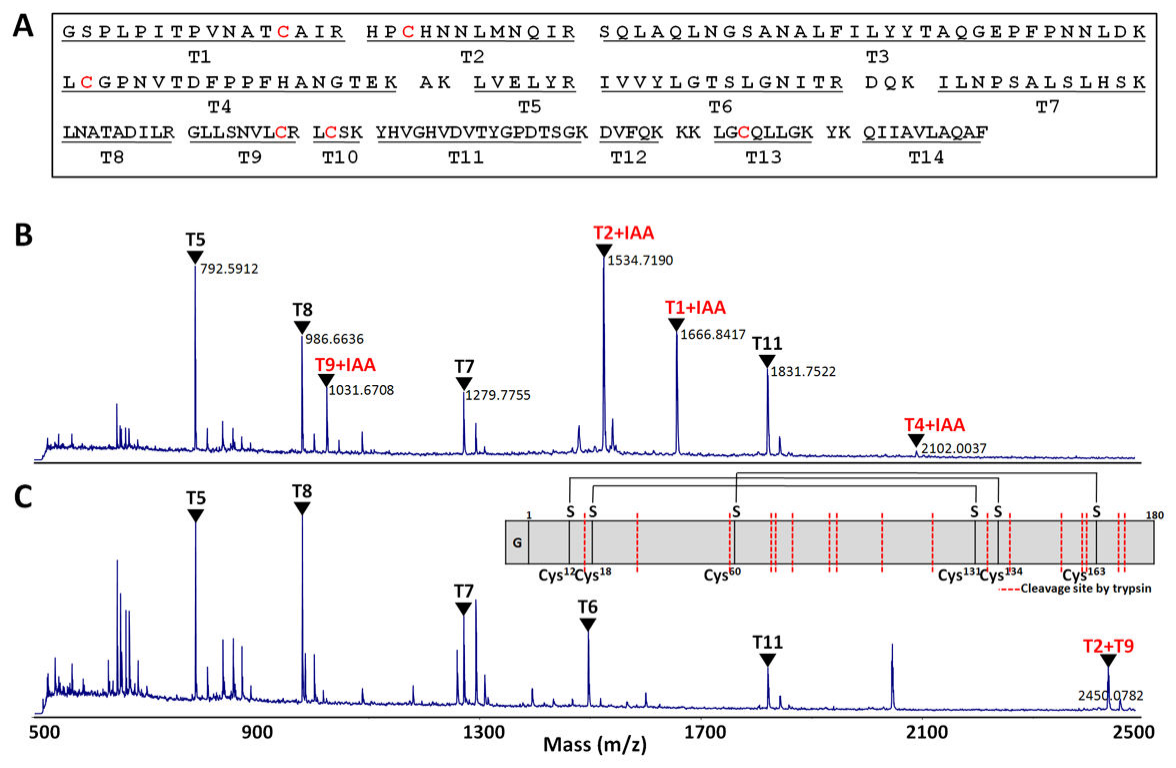
Mass analysis of hLIF purified from *E. coli*. (A) Tryptic peptide map of hLIF (181 aa). Reduced (B) and nonreduced (C) hLIF proteins cleaved from PDIb'a'-hLIF were analyzed using MALDI-TOF MS. Peptide fragments digested by trypsin are shown as red dotted lines on the schematic representation inset.

To confirm the identity and correct disulfide bond formation of purified hLIF [31], MALDI-TOF MS was performed on reduced and nonreduced hLIF obtained from PDIb'a'-hLIF (Figure 4). The MS analysis shows that most of tryptic peptide masses detected correspond to the hLIF fragments that were expected from trypsin digestion (Figure 4). The alkylating agent (IAA) was used to block disulfide bond formation under reducing conditions using DTT. IAA covalently binds to the thiol group of cysteine (Cys). We identified four peptide fragments that had one Cys alkylated by IAA, T1 (Cys12), T2 (Cys18), T4 (Cys60), or T9 (Cys131) (Figure 4B). Two other peptide fragments that contain Cys, T10 (Cys134) and T13 (Cys163), were not detected. Under nonreducing conditions, all peaks that were alkylated with IAA disappeared, and a new peak that corresponds to the exact size (2450 Da) of disulfide-bonded cysteines between T2 and T9 appeared (Figure 4C). The two other disulfide-bonded fragment, T1 + T10 (2,039 Da) and T4 + T13 (2,872 Da), were not detected. MALDI-TOF analysis of reduced and nonreduced hLIF obtained from His8-hLIF demonstrated similar results (data not shown).

### Biological activity

**Figure 5:**
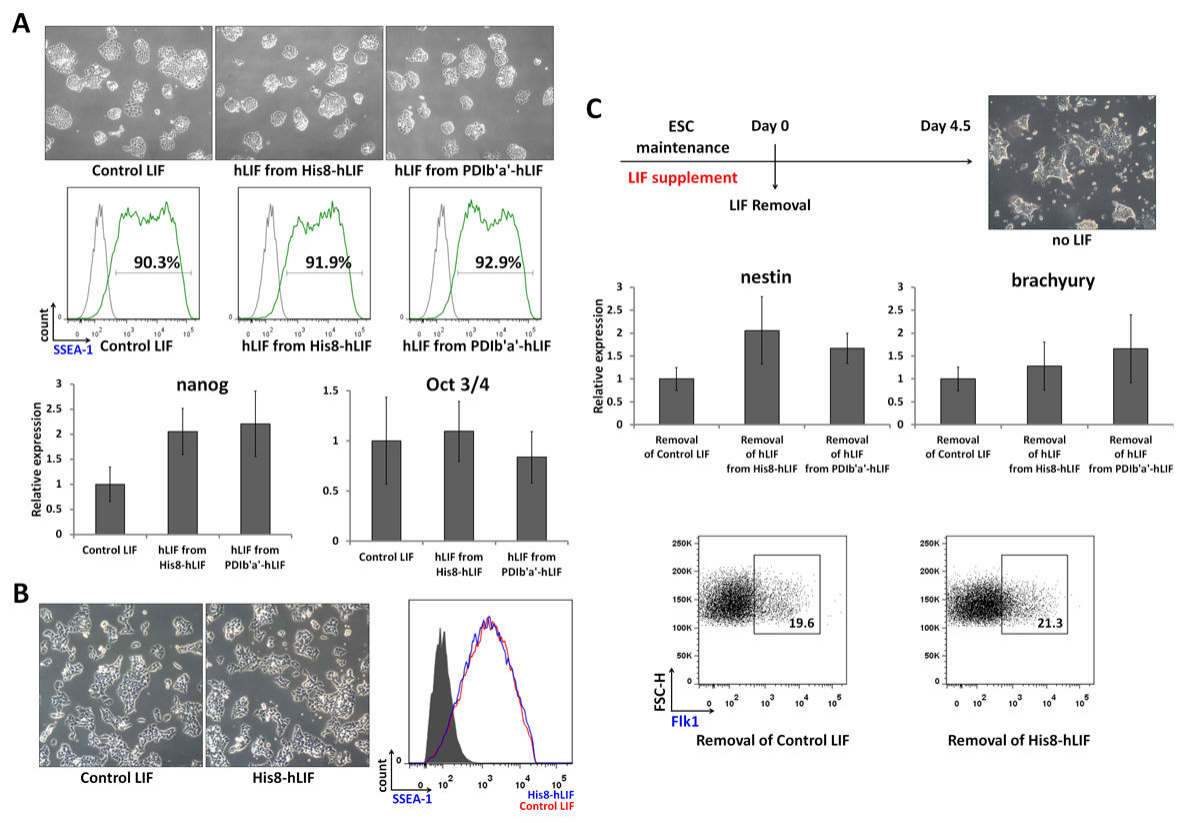
Biological activity. (A) ESCs demonstrated equivalent growth following treatment with control LIF, hLIF from His8-hLIF, and hLIF from hPDIb'a'-hLIF (upper). ESCs demonstrated SSEA-1 expression on the cell surface in the presence of LIFs (middle). The expression levels of two stem cell factors, nanog and Oct3/4, in ESCs treated with LIFs were quantified using qRT-PCR (lower). (B) His8-hLIF-treated cells, like those treated with control LIF, maintained an ESC-like appearance (left and middle). SSEA-1 expression in response to noncleaved His8-hLIF was similar to control LIF (right). (C) Schematic diagram describing ESC maintenance and differentiation processes (upper). Cellular morphology was effected by the withdrawal of LIF (upper right) compared with Figures 5A and 5C. The removal of LIFs induced two differentiation markers, nestin and brachyury, which were expressed at similar or higher levels (middle). ESCs gave rise to Flk-1^+^ mesodermal precursor cells following His8-hLIF treatment (lower).

The biological activities of the purified products were tested by adding proteins to the mESC cultures and comparing their activities to commercially available LIF (Figure 5). No significant differences in activity were observed between the proteins. mESCs grew as well as normal colonies following treatment with purified hLIF obtained from either His8-hLIF or PDIb'a'-hLIF, demonstrating comparable effects with the control LIF (Figure 5A). Purified hLIF maintained SSEA-1 protein surface expression (a marker of stem cells) on mESCs. qRT-PCR analysis demonstrated that the expression of two stem cell factors, nanog and Oct3/4, were also maintained using the purified hLIFs. The average expression level of nanog following treatment with purified LIF was higher than that of the control LIF, but this difference was statistically insignificant. The expression of Oct3/4 was similar to the other three LIFs. Interestingly, noncleaved His8-hLIF also demonstrated the ability to maintain mESCs (Figure 5B). The expression of SSEA-1 on the mESC surface was also maintained following treatment with noncleaved His8-LIF.

The differentiation potential of LIF was examined by omitting LIF supplementation from the culture media (Figure 5C). Non-round cellular morphology with elongated spikes became apparent after 4.5 days without LIF supplementation. The expression levels of two stem cell factors, nanog and Oct3/4, were reduced to < 20% by LIFs removal (data not shown). The removal of control LIF or hLIFs from either His8-hLIF or PDIb'a'-hLIF also allowed nestin and brachyury to be expressed (mesodermal differentiation markers). These two genes demonstrated low expression levels when the cells were cultured with LIF (data not shown). The average expression levels of the differentiation markers were greater following the removal of purified hLIF than the removal of control LIF, but these differences were statistically insignificant (Figure 5C). Another mesodermal precursor, Flk-1 protein, was detected on the cell surface following the removal of noncleaved His8-hLIF or control LIF. About 20% of the cells that were deprived of either control LIF or His8-hLIF were Flk-1^+^. 

## Discussion

Protein misfolding can be an obstacle when attempting to overexpress recombinant proteins in prokaryotes [32]. Misfolding frequently occurs for proteins that contain multiple disulfide bonds since the cytoplasm is a reducing environment that is hostile to disufide bond formation. The hydrophobic core of a protein can be exposed without the proper disulfide bond formation, causing the misfolded proteins to aggregate and form IBs. LIF has three disulfide bonds that may contribute to its insolubility during overexpression in *E. coli* (Figure 2A). Two protein tags, Trx and GST, have previously been introduced to increase the solubility of overexpressed hLIF in *E. coli* [17-21]. Trx, a bacterial redox protein, is believed to effect disulfide bond formation. However, the molecular mechanism explaining how GST increases solubility remains unknown.

Proper disulfide bonds are crucial for protein folding and function. In eukaryotes, disulfide bonds are formed in the endoplasmic reticulum with the help of chaperone proteins (such as PDI) that increase the rate of disulfide bond formation (oxidation) and rearrangement (isomerization) [33]. PDI increases the soluble production of disulfide bond-containing proteins in the cytoplasm [34] and periplasm [35] of *E. coli*. Therefore, we used PDI as a fusion protein for hLIF and observed that this enzyme increases the solubility of hLIF from 22% to 97% (Figure 2A and Table 1). One problem with using PDI as a fusion protein is its large size (57 kDa). The enzyme is comprised of four thioredoxin domains—a, b, b', and a'—followed by an extension denoted as “c” that is responsible for endoplasmic reticulum retention signaling [33]. The a and the a' domains each have one catalytically active CGHC motif, and the b and the b' domains are redox inactive but still demonstrate chaperone activities. The b'a' domain (PDIb'a') is about half the size of the whole protein and demonstrates relatively strong PDI activities. We successfully developed PDIb'a' for use as a molecular chaperone of fibroblast growth factor 2 (FGF2) expression in *E. coli* [23]. Here, we attempted to use PDIb'a' as a fusion tag for hLIF. Even though PDIb'a' increased the solubility of hLIF, more than half remained insoluble, in contrast to the case for FGF2 (Figure 2A and Table 1).

Low temperature expression has been successfully used to increase the solubility of several proteins in *E. coli* [19,36-40]. When we decreased the expression temperature, His8- and PDIb'a'-tagged hLIF demonstrated dramatically improved solubilities (Fig. 2B and Table 1). It is possible that lowering the temperature might shift codon usage enough to alleviate some codon usage-associated expression problems [41]. In addition, slow protein expression may allow enough time for the nascent peptide chains to fold properly and/or to stabilize one or more important folding intermediates [42-46]. However, the molecular mechanisms that allow slow temperature to increase protein solubility are not still completely understood and probably vary from case to case.

When a buffer with a low salt concentration was used, His8-hLIF precipitated (Table S2 in File SI). However, once the hLIF tags were removed the protein did not precipitate at the reduced salt concentrations. The extra 19 amino acids (a result of Gateway plasmid recombination; Figure 1B) and the His8 tag seem to make the protein unstable at low salt concentrations. The advantages of using Gateway technology include easy screening for protein overexpression among the various tagging systems and the rapid and highly efficient transfer of entry vectors into various destination vectors without the need for PCR; however, the disadvantage is the addition of 19 amino acid sequences between the tags and the TEV cleavage site. These extra amino acids may affect the stability or function of the fusion proteins, especially if the host protein or its co-tags are relatively small. The pIs of the tags were quite different when calculated using ExPASy (http://expasy.org). The His8, PDI, and PDIb'a' tags have predicted pIs of 10.3, 4.7 and 4.6, respectively, and the pIs of His8-hLIF, PDI-hLIF and PDIb'a'-hLIF are calculated to be 9.3, 5.7, and 6.0, respectively, suggesting that charge may have an influence on solubility at low salt concentrations. The long-term solubility of both His8-hLIF and hLIF cleaved from His8-hLIF were better than PDIb'a'-hLIF and hLIF cleaved from PDIb'a'-hLIF. This could be due to the isomerase activity of PDIb'a' that can make properly folded hLIF unstable. The fact that hLIF cleaved from PDIb'a'-hLIF is also unstable suggests that PDIb'a' might be a slight contaminant in the purified hLIF. 

The 27 N-terminal amino acids, which include the octahistidine tag and the extra 19 amino acids, did not affect the biological activity of His8-hLIF. The three types of hLIF, His8-hLIF; hLIF cleaved form His8-hLIF; and hLIF cleaved from PDIb'a'-hLIF, were equally functional when added to mESCs (Figure 5). This is consistent with the finding that the His6 and Trx-His6 tags are also functional [19]. The N-terminus of LIF is far from the receptor binding site and seems to be flexible [47]. Therefore, extra tags at the N-terminus of LIF do not interfere with the binding of the ligand to its receptor. 

There are six Cys residues in hLIF, and together they participate in formation of the three intramolecular disulfide bonds [31,48]. From the combination of reduced and nonreduced hLIF samples, MS analysis detected four Cys-containing fragments and one disulfide bond fragment. The other possible two Cys-containing fragments and two disulfide-bonded fragment were not detected (Figure 4). This does not necessarily mean that the two of disulfide bonds are not formed by the purified hLIF. Often peptide fragments are not detected for a variety of reasons, such as the physical properties of the peptide, the compatibility of the matrix with the peptide and diagnostic sensitivity. It is likely that the three correct disulfide bonds were intact, since all four Cys-containing fragments detected under reduced conditions disappeared under nonreducing conditions. Above all, the purified hLIF demonstrated biological activity similar to control LIF (Figure 5), suggesting that it adopts a native conformation that includes the three correct disulfide bonds. 

Although the hLIF yield from PDIb'a'-hLIF was occasionally better than His8-hLIF, the average hLIF yield from PDIb'a'-hLIF was slightly less than His8-hLIF. The hLIF yield from PDIb'a'-hLIF can be potentially improved in future scale-up and optimization trials since PDIb'a'-hLIF is more stable than His8-hLIF under various buffer conditions (Table S2 in File SI); therefore, additional expression and purification options can be attempted. Our current yield is not better than those from previous studies that used bacterial expression systems. One study reported a very high yield (> 100 mg/l) [19]. However, that study did not cleave His6 or Trx-His6 tags, so these fusion proteins may not be suitable for medical applications since they may invoke immune responses during *in vivo* applications. In addition, the endotoxin levels were not reported, which is also a critical factor for *in vivo* applications. Other studies either did not report the endotoxin levels or reported high levels [15,18].

hLIF has five N-linked glycosylation sites [48]. Since *E. coli* has no machinery for protein glycosylation, hLIF from *E. coli* is nonglycosylated. Often, glycosylation increases the half-life of proteins circulating in the blood by protecting them from proteases without effecting protein receptor binding. This is consistent with our results demonstrating that both purified hLIF from *E. coli* and control LIF from mammalian cells can successfully maintain the pluripotency of ESC at a concentration of 1 nM (Figure 5). Glycosylation-like modifications, such as PEGylation and chitosanylation, can be performed for future *in vivo* applications to increase half-life of this bacterially expressed hLIF.

## Conclusion

Here, we have improved the solubility of hLIF in *E. coli* by fusing it with protein disulphide isomerase and have established purification methods for octahistidine-tagged hLIF and PDIb'a'-hLIF fusion proteins. The purified hLIF was highly pure and free of endotoxins. We identified intramolecular disulfide bonds using mass spectroscopy. The proteins purified from both constructs were equally effective at maintaining the pluripotency of mESCs. We also found that the 27 N-terminal amino acids (additional 19 amino acids at the N-terminus of hLIF from Gateway system and the octahistidine tag) made hLIF unstable with regards to solubility at low salt concentrations, however this did not affect the biological activity.

## Supporting Information

File S1 **Supporting figures and tables**.
**Table S1**. **Percentage His8-hLIF cleavage using TEV protease**. **Table S2**. **Overnight precipitation of His8-hLIF and PDIb'a'-hLIF under various buffer conditions**. **Figure S1**. **Codon-optimized DNA sequences of (A) hLIF, (B) PDI, (C) His8-hLIF and (D) PDI-hLIF for expression in *E. coli***. PDIb'a' and TEV recognition sites are shown in blue and red, respectively. **Figure S2**. **His8-hLIF cleavage using TEV protease**. The following conditions were tested; protein:TEV ratio of 15:1, 5:1 and 1:1 at RT, 18°C, and 30°C in the case of His8-hLIF. Various amounts of TEV were used to treat His8-hLIF overnight at RT, 18°C or 30°C at 15:1 His8-hLIF:TEV (w/w; left panel), 5:1 His8-hLIF:TEV (w/w; middle panel), or 1:1 His8-hLIF:TEV (w/w; right panel). Reactions were performed in 200 mM NaCl and 400 mM imidazole (upper panel) or 500 mM NaCl and 1 M imidazole (lower panel). The fusion protein was cleaved almost completely at 1:1 protein:TEV (w/w). Based on this result, PDIb'a'-hLIF was first treated overnight with 10:1 protein:TEV (w/w) at RT. Since PDIb'a'-hLIF was almost completely cleaved under these conditions, no further optimization was carried out. **Figure S3**. **Long-term stability of His8-hLIF, PDIb'a'-hLIF, and hLIF derived from the fusion proteins**. His8-hLIF and PDIb'a'-hLIF were incubated at 37°C for various lengths of time. The purified hLIFs derived from the tagged proteins were kept at room temperature for various lengths of time. The protein supernatants were separated from the precipitate by centrifugation at 16,000 x g for 1 minute, and the supernatant concentration was determined using the Bradford method. ○, hLIF from PDIb'a'-hLIF (buffer D; 20 mM Tris-HCl, pH 8.0, 300 mM NaCl, 5% glycerol (v/v), 100 mM imidazole); □, PDIb'a'-hLIF (buffer G; 20 mM Tris-HCl, pH 8.0, 5% glycerol (v/v), 0.5 mM EDTA); ●, hLIF from His8-hLIF (buffer D); ■, His8-hLIF (buffer C; 20 mM Tris-HCl, pH 8.0, 300 mM NaCl, 5% glycerol (v/v), 1 M imidazole).
(DOCX)
