# Correction: Soluble Expression of Human Leukemia Inhibitory Factor with Protein Disulfide Isomerase in *Escherichia coli* and Its Simple Purification

**DOI:** 10.1371/annotation/4b2acdb5-5bab-4bca-8fbc-f9f293b38ee0

**Published:** 2014-01-06

**Authors:** Jung-A Song, Bon-Kyung Koo, Seon-Ha Chong, Kyunhoo Kim, Dong Kyu Choi, Thu Trang Thi Vu, Minh Tan Nguyen, Boram Jeong, Han-Bong Ryu, Injune Kim, Yeon Jin Jang, Robert Charles Robinson, Han Choe

The name of the first author is incorrectly given. The correct name is: Jung-A Song. 

The correct Citation is: 

Song J-A, Koo B-K, Chong S-H, Kim K, Choi DK, et al. (2013) Soluble Expression of Human Leukemia Inhibitory Factor with Protein Disulfide Isomerase in Escherichia coli and Its Simple Purification. PLoS ONE 8(12): e83781. doi:10.1371/journal.pone.0083781.

