# Supplementary material for: Soluble Expression of Human Leukemia Inhibitory Factor with Protein Disulfide Isomerase in Escherichia coli and Its Simple Purification
Source: PLoS One. 2013 Dec 16;8(12):e83781. doi: 10.1371/journal.pone.0083781 (PMC3865251; doi:10.1371/journal.pone.0083781)
Supplement: File S1 — Supporting figures and tables. Table S1. Percentage His8-hLIF cleavage using TEV protease. Table S2. Overnight precipitation of His8-hLIF and PDIb'a'-hLIF under various buffer conditions. Figure S1. Codon-optimized DNA sequences of (A) hLIF, (B) PDI, (C) His8-hLIF and (D) PDI-hLIF for expression in E. coli. PDIb'a' and TEV recognition sites are shown in blue and red, respectively. Figure S2. His8-hLIF cleavage using TEV protease. The following conditions were tested; protein:TEV ratio of 15:1, 5:1 and 1:1 at RT, 18°C, and 30°C in the case of His8-hLIF. Various amounts of TEV were used to treat His8-hLIF overnight at RT, 18°C or 30°C at 15:1 His8-hLIF:TEV (w/w; left panel), 5:1 His8-hLIF:TEV (w/w; middle panel), or 1:1 His8-hLIF:TEV (w/w; right panel). Reactions were performed in 200 mM NaCl and 400 mM imidazole (upper panel) or 500 mM NaCl and 1 M imidazole (lower panel). The fusion protein was cleaved almost completely at 1:1 protein:TEV (w/w). Based on this result, PDIb'a'-hLIF was first treated overnight with 10:1 protein:TEV (w/w) at RT. Since PDIb'a'-hLIF was almost completely cleaved under these conditions, no further optimization was carried out. Figure S3. Long-term stability of His8-hLIF, PDIb'a'-hLIF, and hLIF derived from the fusion proteins. His8-hLIF and PDIb'a'-hLIF were incubated at 37°C for various lengths of time. The purified hLIFs derived from the tagged proteins were kept at room temperature for various lengths of time. The protein supernatants were separated from the precipitate by centrifugation at 16,000 x g for 1 minute, and the supernatant concentration was determined using the Bradford method. ○, hLIF from PDIb'a'-hLIF (buffer D; 20 mM Tris-HCl, pH 8.0, 300 mM NaCl, 5% glycerol (v/v), 100 mM imidazole); □, PDIb'a'-hLIF (buffer G; 20 mM Tris-HCl, pH 8.0, 5% glycerol (v/v), 0.5 mM EDTA); ●, hLIF from His8-hLIF (buffer D); ■, His8-hLIF (buffer C; 20 mM Tris-HCl, pH 8.0, 300 mM NaCl, 5% glycerol (v/v), 1 M imidazole). (DOCX) [file pone.0083781.s001.docx]

**Supplementary Information**

**Table S1. Percentage His8-hLIF cleavage using TEV protease**

|  | NaCl | Imidazole | Temperature | Protein:TEV protease (w/w) | | |
| --- | --- | --- | --- | --- | --- | --- |
|  | (mM) | (mM) | (^o^C) | 15:1 | 5:1 | 1:1 |
| Cleavage by TEV protease | 200 | 400 | RT | 21% | 63% | - |
|  |  |  | 18 | 32% | 68% | - |
|  |  |  | 30 | - | - | 95% |
|  | 500 | 1000 | RT | 41% | 60% | - |
|  |  |  | 18 | 31% | 30% | - |

**Table S2. Overnight precipitation of His8-hLIF and PDIb'a'-hLIF under various buffer conditions**

|  | NaCl  (mM) | Tris  (mM) | pH | EDTA  (mM) | DTT  (mM) | Glycerol  (%) | Glutamate  (mM) | β-ME  (mM) | Precipitation  (%) |
| --- | --- | --- | --- | --- | --- | --- | --- | --- | --- |
| PDIb'a'-hLIF | 300 | 20 | 8.0 | - | - | 5 | **-** | **-** | Negligible |
|  | - | 20 | 8.0 | - | - | 5 | **-** | **-** | 9 |
| His8-hLIF | 300 | 20 | 8.0 |  |  | 5 |  |  | Negligible |
|  | - | 20 | 8.0 | - | - | 5 | **-** | **-** | 94 |
| His8-hLIF | 100 | 20 | 7.0 | 10 | - | 5 | - | - | 95 |
|  | 100 | 20 | 7.0 | 10 | 1 | 5 | - | - | 92 |
|  | 150 | 20 | 7.3 | 10 | 1 | 10 | - | - | 53 |
|  | 100 | 20 | 7.3 | 10 | 1 | 10 | 100 | - | 53 |
|  | 500 | 20 | 7.5 | 10 | - | 5 | - | 5 | 43 |

**Figure S1. Codon-optimized DNA sequences of (A) hLIF, (B) PDI, (C) His8-hLIF and (D) PDI-hLIF for expression in *E. coli*.** PDIb'a' and TEV recognition sites are shown in blue and red, respectively.

(A) 5'-agtcctctgccgatcacacctgtgaatgccacatgcgcaatccgtcatccttgccacaataatctgatgaatcagatccgttcacagctcgcgcagctgaatggatcagctaacgcactttttattctgtattataccgcacagggagagccatttccgaacaacctggataaattgtgtggcccgaacgtaactgattttccgccattccatgctaatgggacggaaaaggccaagctggttgaactgtatcgtattgttgtttacctgggcacctctctgggcaacattactcgcgatcagaaaattctgaatccgagcgcactgagtctccactctaaactgaacgccacagctgacatattacggggtttactgagcaatgtgttgtgtcgcctgtgttcgaaatatcatgtaggtcacgtggatgtcacctacgggcccgacacgtccggtaaagatgtctttcaaaaaaaaaaattgggctgccaactgctgggcaagtataaacagataattgcggttctggcccaggcgttctga-3'

(translation=splpitpvnatcairhpchnnlmnqirsqlaqlngsanalfilyytaqgepfpnnldklcgpnvtdfppfhangtekaklvelyrivvylgtslgnitrdqkilnpsalslhsklnatadilrgllsnvlcrlcskyhvghvdvtygpdtsgkdvfqkkklgcqllgkykqiiavlaqaf)

(B)

5'-gacgcaccggaagaagaggatcatgtcctggttctgcgcaaaagcaacttcgcggaagcactggccgcacataaatatctgctggtggaattttatgctccttggtgcggtcattgcaaagccctggccccggagtacgccaaagccgcaggtaaactgaaagctgaaggtagcgaaatccgcctggcaaaggttgatgctacggaagagagtgacctggcgcagcagtatggtgtccgcggctatcctacaattaaattcttccgtaacggtgataccgcatctccaaaagaatataccgctggtcgcgaggcggacgatattgttaactggctgaagaaacgcactggccctgccgcaaccaccctgcctgatggcgctgctgccgaaagcctggtcgaaagtagcgaagttgccgtcattggtttctttaaggatgtagaatctgacagtgccaaacagtttctgcaagcggcagaggctatcgatgacatcccgttcggcatcacctctaacagtgacgtattcagtaaataccaactggataaagacggcgttgtgctgttcaagaaatttgatgaaggtcgcaacaactttgagggtgaggtgaccaaggagaacctgctggattttattaagcacaaccaactgccgctggttattgaatttacagaacaaacggcgccgaaaattttcggcggtgagattaaaacacatatcctgctgtttctgccgaagagcgtttctgattacgatggtaaactgagtaattttaaaaccgccgcagaatctttcaaaggtaagattctgttcattttcattgatagcgaccacacggacaatcagcgtatcctggagttctttggtctgaagaaagaggaatgcccggctgtgcgtctgattacgctggaagaggaaatgacaaagtacaagccggagagcgaggaactgactgcagaacgtatcaccgaattttgtcatcgtttcctggaggggaagattaagccgcatctgatgagccaggaactgccggaggattgggacaaacagccagtgaaagttctggtggggaagaattttgaagatgtggccttcgatgagaagaagaatgtgtttgtggagttctacgccccgtggtgtgggcactgtaaacagctggcgccgatctgggacaaactgggcgaaacgtataaagatcacgaaaatattgtgatcgcgaaaatggattctaccgcgaatgaagtagaagcggtaaaagtacactcttttccgacgctgaaattctttccagcgagcgcggatcgtactgtcattgattataatggcgaacgtactctggacggctttaagaaatttctggaaagcggcggccaggatggcgcgggcgatgatgatgacctggaagacctggaagaagcggaggaaccagacatggaggaggatgacgaccagaaagcggtc-3'

(translation=DAPEEEDHVLVLRKSNFAEALAAHKYLLVEFYAPWCGHCKALAPEYAKAA

GKLKAEGSEIRLAKVDATEESDLAQQYGVRGYPTIKFFRNGDTASPKEYTAGREADDIVNWLKKRTGPAATTLPDGAAAESLVESSEVAVIGFFKDVESDSAKQFLQAAEAIDDIPFGITSNSDVFSKYQLDKDGVVLFKKFDEGRNNFEGEVTKENLLDFIKHNQLPLVIEFTEQTAPKIFGGEIKTHILLFLPKSVSDYDGKLSNFKTAAESFKGKILFIFIDSDHTDNQRILEFFGLKKEECPAVRLITLEEEMTKYKPESEELTAERITEFCHRFLEGKIKPHLMSQELPEDWDKQPVKVLVGKNFEDVAFDEKKNVFVEFYAPWCGHCKQLAPIWDKLGETYKDHENIVIAKMDSTANEVEAVKVHSFPTLKFFPASADRTVIDYNGERTLDGFKKFLESGGQDGAGDDDDLEDLEEAEEPDMEEDDDQKAV)

(C) 5'-**atgaaa**caccaccaccaccaccaccaccac**ggtggtctggttccgcgtggttccatcacaagtttgtacaaaaaagcaggcttc**atgcaccatcatcatcaccatcatcatgaaaatctgtactttcaa**ggt**agtcctctgccgatcacacctgtgaatgccacatgcgcaatccgtcatccttgccacaataatctgatgaatcagatccgttcacagctcgcgcagctgaatggatcagctaacgcactttttattctgtattataccgcacagggagagccatttccgaacaacctggataaattgtgtggcccgaacgtaactgattttccgccattccatgctaatgggacggaaaaggccaagctggttgaactgtatcgtattgttgtttacctgggcacctctctgggcaacattactcgcgatcagaaaattctgaatccgagcgcactgagtctccactctaaactgaacgccacagctgacatattacggggtttactgagcaatgtgttgtgtcgcctgtgttcgaaatatcatgtaggtcacgtggatgtcacctacgggcccgacacgtccggtaaagatgtctttcaaaaaaaaaaattgggctgccaactgctgggcaagtataaacagataattgcggttctggcccaggcgttc-3'

(translation=**MK**HHHHHHHH**GGLVPRGSITSLYKKAGF**MHHHHHHHHENLYFQ**G**SPLPITPVNATCAIRHPCHNNLMNQIRSQLAQLNGSANALFILYYTAQGEPFPNNLDKLCGPNVTDFPPFHANGTEKAKLVELYRIVVYLGTSLGNITRDQKILNPSALSLHSKLNATADILRGLLSNVLCRLCSKYHVGHVDVTYGPDTSGKDVFQKKKLGCQLLGKYKQIIAVLAQAF)

(D) 5'-**atgaaa**caccatcaccatcaccatcaccat**gagctc**gacgcaccggaagaagaggatcatgtcctggttctgcgcaaaagcaacttcgcggaagcactggccgcacataaatatctgctggtggaattttatgctccttggtgcggtcattgcaaagccctggccccggagtacgccaaagccgcaggtaaactgaaagctgaaggtagcgaaatccgcctggcaaaggttgatgctacggaagagagtgacctggcgcagcagtatggtgtccgcggctatcctacaattaaattcttccgtaacggtgataccgcatctccaaaagaatataccgctggtcgcgaggcggacgatattgttaactggctgaagaaacgcactggccctgccgcaaccaccctgcctgatggcgctgctgccgaaagcctggtcgaaagtagcgaagttgccgtcattggtttctttaaggatgtagaatctgacagtgccaaacagtttctgcaagcggcagaggctatcgatgacatcccgttcggcatcacctctaacagtgacgtattcagtaaataccaactggataaagacggcgttgtgctgttcaagaaatttgatgaaggtcgcaacaactttgagggtgaggtgaccaaggagaacctgctggattttattaagcacaaccaactgccgctggttattgaatttacagaacaaacggcgccgaaaattttcggcggtgagattaaaacacatatcctgctgtttctgccgaagagcgtttctgattacgatggtaaactgagtaattttaaaaccgccgcagaatctttcaaaggtaagattctgttcattttcattgatagcgaccacacggacaatcagcgtatcctggagttctttggtctgaagaaagaggaatgcccggctgtgcgtctgattacgctggaagaggaaatgacaaagtacaagccggagagcgaggaactgactgcagaacgtatcaccgaattttgtcatcgtttcctggaggggaagattaagccgcatctgatgagccaggaactgccggaggattgggacaaacagccagtgaaagttctggtggggaagaattttgaagatgtggccttcgatgagaagaagaatgtgtttgtggagttctacgccccgtggtgtgggcactgtaaacagctggcgccgatctgggacaaactgggcgaaacgtataaagatcacgaaaatattgtgatcgcgaaaatggattctaccgcgaatgaagtagaagcggtaaaagtacactcttttccgacgctgaaattctttccagcgagcgcggatcgtactgtcattgattataatggcgaacgtactctggacggctttaagaaatttctggaaagcggcggccaggatggcgcgggcgatgatgatgacctggaagacctggaagaagcggaggaaccagacatggaggaggatgacgaccagaaagcggtc**ggtaccggatcttacatcacaagtttgtacaaaaaagcaggcttc**atgcaccatcatcatcaccatcatcatgaaaatctgtactttcaa**ggt**agtcctctgccgatcacacctgtgaatgccacatgcgcaatccgtcatccttgccacaataatctgatgaatcagatccgttcacagctcgcgcagctgaatggatcagctaacgcactttttattctgtattataccgcacagggagagccatttccgaacaacctggataaattgtgtggcccgaacgtaactgattttccgccattccatgctaatgggacggaaaaggccaagctggttgaactgtatcgtattgttgtttacctgggcacctctctgggcaacattactcgcgatcagaaaattctgaatccgagcgcactgagtctccactctaaactgaacgccacagctgacatattacggggtttactgagcaatgtgttgtgtcgcctgtgttcgaaatatcatgtaggtcacgtggatgtcacctacgggcccgacacgtccggtaaagatgtctttcaaaaaaaaaaattgggctgccaactgctgggcaagtataaacagataattgcggttctggcccaggcgttc-3'

(translation=**MK**HHHHHHHH**EL**DAPEEEDHVLVLRKSNFAEALAAHKYLLVEFYAPWCGHCKALAPEYAKAAGKLKAEGSEIRLAKVDATEESDLAQQYGVRGYPTIKFFRNGDTASPKEYTAGREADDIVNWLKKRTGPAATTLPDGAAAESLVESSEVAVIGFFKDVESDSAKQFLQAAEAIDDIPFGITSNSDVFSKYQLDKDGVVLFKKFDEGRNNFEGEVTKENLLDFIKHNQLPLVIEFTEQTAPKIFGGEIKTHILLFLPKSVSDYDGKLSNFKTAAESFKGKILFIFIDSDHTDNQRILEFFGLKKEECPAVRLITLEEEMTKYKPESEELTAERITEFCHRFLEGKIKPHLMSQELPEDWDKQPVKVLVGKNFEDVAFDEKKNVFVEFYAPWCGHCKQLAPIWDKLGETYKDHENIVIAKMDSTANEVEAVKVHSFPTLKFFPASADRTVIDYNGERTLDGFKKFLESGGQDGAGDDDDLEDLEEAEEPDMEEDDDQKAV**GTGSYITSLYKKAGF**MHHHHHHHHENLYFQ**G**SPLPITPVNATCAIRHPCHNNLMNQIRSQLAQLNGSANALFILYYTAQGEPFPNNLDKLCGPNVTDFPPFHANGTEKAKLVELYRIVVYLGTSLGNITRDQKILNPSALSLHSKLNATADILRGLLSNVLCRLCSKYHVGHVDVTYGPDTSGKDVFQKKKLGCQLLGKYKQIIAVLAQAF)

**Figure S2. His8-hLIF cleavage using TEV protease.** The following conditions were tested; protein:TEV ratio of 15:1, 5:1 and 1:1 at RT, 18°C, and 30°C in the case of His8-hLIF. Various amounts of TEV were used to treat His8-hLIF overnight at RT, 18°C or 30°C at 15:1 His8-hLIF:TEV (w/w; left panel), 5:1 His8-hLIF:TEV (w/w; middle panel), or 1:1 His8-hLIF:TEV (w/w; right panel). Reactions were performed in 200 mM NaCl and 400 mM imidazole (upper panel) or 500 mM NaCl and 1 M imidazole (lower panel). The fusion protein was cleaved almost completely at 1:1 protein:TEV (w/w). Based on this result, PDIb'a'-hLIF was first treated overnight with 10:1 protein:TEV (w/w) at RT. Since PDIb'a'-hLIF was almost completely cleaved under these conditions, no further optimization was carried out.


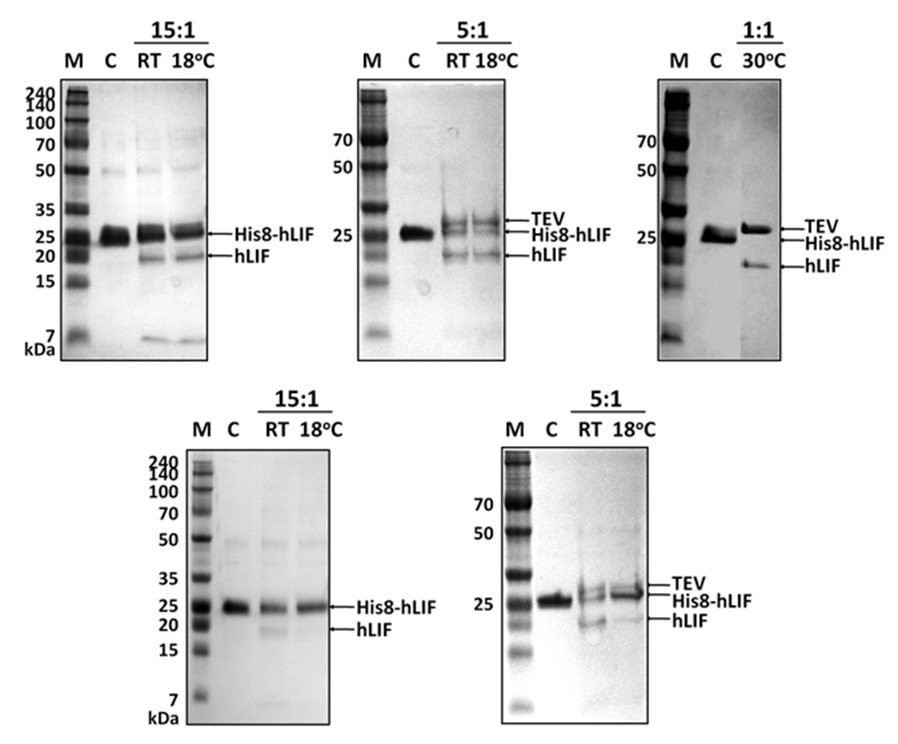


**Figure S3. Long-term stability of His8-hLIF, PDIb'a'-hLIF, and hLIF derived from the fusion proteins.** His8-hLIF and PDIb'a'-hLIF were incubated at 37°C for various lengths of time. The purified hLIFs derived from the tagged proteins were kept at room temperature for various lengths of time. The protein supernatants were separated from the precipitate by centrifugation at 16,000 x g for 1 minute, and the supernatant concentration was determined using the Bradford method. ○, hLIF from PDIb'a'-hLIF (buffer D; 20 mM Tris-HCl, pH 8.0, 300 mM NaCl, 5% glycerol (v/v), 100 mM imidazole); □, PDIb'a'-hLIF (buffer G; 20 mM Tris-HCl, pH 8.0, 5% glycerol (v/v), 0.5 mM EDTA); ●, hLIF from His8-hLIF (buffer D); ■, His8-hLIF (buffer C; 20 mM Tris-HCl, pH 8.0, 300 mM NaCl, 5% glycerol (v/v), 1 M imidazole).**
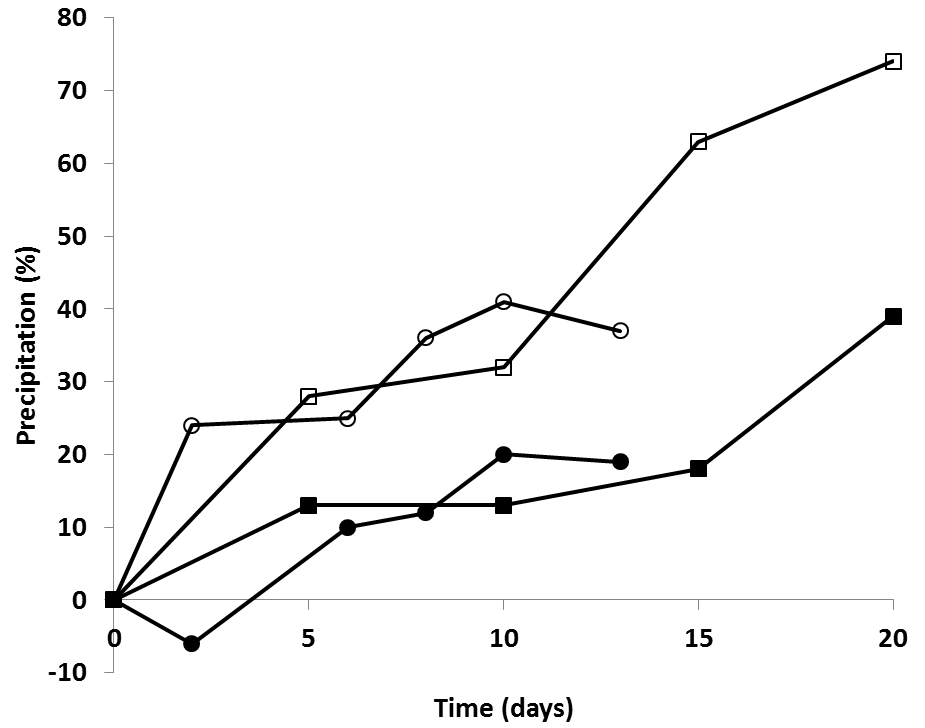
**
